# Prejudice against and desired social distance from refugees, people with mental illness and patients with COVID-19 in athens

**DOI:** 10.1192/j.eurpsy.2021.338

**Published:** 2021-08-13

**Authors:** L.E. Peppou, A. Bechraki, G. Petraki, M. Marouga, D. Mareta, K. Kontoangelos, M. Economou

**Affiliations:** 1 Unit Of Social Psychiatry & Psychosocial Care, University Mental Health, Neurosciences and Precision Medicine Research Institute “Costas Stefanis” (UMHRI), Athens, Greece; 2 First Department Of Psychiatry, National & Kapodistrian University of Athens, Athens, Greece

**Keywords:** social stigma, Refugees, Mental illness, coronavirus

## Abstract

**Introduction:**

Stigma is omnipresent in human societies, both globally and historically; while it is also discerned in other primates. On these grounds, it has been suggested to be the product of natural selection and therefore to protect against threats to effective group functioning. Nonetheless, in contemporary society, stigma raises fundamental ethical concerns, while it actually impinges on public health

**Objectives:**

To explore prejudicial attitudes and desired social distance from recovered COVID-19 patients, people with mental illness and refugees in Athens region.

**Methods:**

A convenience sample of 360 residents of Athens region participated in the study, after being recruited from social media. The questionnaire was distributed online and encompassed: i) the Prejudicial Attitudes Survey, (ii) the Social Distance scale, (iii) the Interpersonal Reactivity Index and information about respondents’ socio-demographic characteristics and personal experience with the three population subgroups. The stigma measures were included three times, one for each out-group.

**Results:**

Repeated ANOVA revealed that negative attitudes were predominantly expressed for refugees. On the contrary, positive attitudes were predominantly expressed for people with mental illness. Interestingly, desired social distance was greater from people with mental illness (mean = 32.37) compared to refugees (mean = 25.47) and recovered COVID-19 patients (mean = 24.17).

**Conclusions:**

Stigma towards people with mental illness and refugees is still prevalent in Greece. Anti-stigma efforts should target prejudices in the case of refugees and social distance in the case of mental illness. To date, no stigma attached to COVID-19 has been discerned in the country

**Disclosure:**

No significant relationships.

